# Digital Innovation in Healthcare: A Device with A Method for Monitoring, Managing and Preventing the Risk of Chronic Polypathological Patients

**Published:** 2020-02-20

**Authors:** G Improta, V De Luca, M Illario, M Triassi

**Affiliations:** 1 Department of Public Health, School of Medicine and Surgery, University of Naples “Federico II”, Naples, Italy; 2 Research and Development Unit, Federico II University Hospital, Naples, Italy; 3 Health Innovation Division, General Directorate for Health, Campania Region, Naples, Italy

**Keywords:** digital innovation, clinical decision-making, chronic patients

## Abstract

New digital technologies can have a huge impact on the traditional healthcare sector, both from a clinical and economic perspective. Doctors and health specialists will increasingly need technology to improve the services they provide to their patients. Here a novel patented device for automatic processing of clinical data of chronic poly-pathological patients is presented. The invention consists of a reconfigurable equipment that allows the assessment of clinical risk severity indexes that can be customized for polypathological patients and which acts both as a decision support system for specialist doctors in the diagnosis and treatment phases, and as a monitoring system in the clinical environment.

## I. INTRODUCTION

Digital technologies have successfully transformed most sectors of the economy, ranging from finance to entertainment, with a significant impact on healthcare. The huge investments in digital health technologies by start-ups and established technological companies aim in fact at a radical transformation of health services.

Although the healthcare sector is complex and the introduction of technological and digital innovations requires important “change management” work so that the technological solutions are integrated into the assistance processes and adopted in clinical practice, it is becoming increasingly dependent on digital technologies to improve patient-specialist communication, provide efficient clinical treatments, optimize the diagnosis of dangerous diseases, reduce threats to public health and optimize health services globally. The future of the health sector is therefore deeply tied to technological progress.

New technologies will continue to change the traditional healthcare sector as we know it, influencing this market from different perspectives. On the one hand, it will have an impact on the commercial side of the sector, which means that companies and organizations will be able to more precisely deliver optimized services to customers and increase profitability. On the other hand, it will influence the services provided by industry by discovering new ways of treating diseases, optimizing diagnoses and improving delicate decision-making processes. Doctors and health specialists will increasingly need technology to improve the services they provide to their patients. Technological and digital innovation will help doctors in their different fields, from improving accuracy during the execution of a complicated operation, to optimizing a diagnosis using intelligent systems capable of understanding health problems and complicated diseases.

Among the most innovative digital tools in the field of healthcare, the use of artificial intelligence plays a fundamental role. In the medical field, artificial intelligence is based on the analysis and interpretation of huge quantities of data sets in order to help doctors make better decisions, effectively manage information on patient data, create personalized medicine plans from complex data sets and discover new drugs. Among the applications of artificial intelligence, we find:

- Clinical decision support: artificial intelligence proves useful in the context of clinical decision support to help doctors make better decisions faster with the recognition of patterns of health complications recorded much more accurately than the human brain. Time saved and diagnosed conditions are critical in an area where time spent and decisions made can change patients’ lives.
- Information management: artificial intelligence in healthcare is an excellent addition to information management for both the doctor and the patient. With patients turning to doctors faster or not at all when using telemedicine, valuable time and money are saved, eliminating the effort of healthcare professionals and increasing patient comfort.
- Discovery of new drugs: health and pharmaceutical companies currently exploit artificial intelligence as a support tool for the discovery of new drugs to optimize the long timescales and processes related to the discovery and approval of a new drug, from clinical tests to its placing on the market.

The use of artificial intelligence to support clinical decisions, in particular, is the subject of a recent patent developed developed at the Public Health Department of the School of Medicine and Surgery of the Federico II University of Naples by: Prof. Maria Triassi (deputy director of the Department), Dr. Giovanni Improta (researcher of the Department), Dr. Vincenzo Abate (then postdoctoral fellow of the Department), Dr. Mario Alessandro Russo (PhD student of the Department), Prof. Stefania Santini (Associate Professor of DIETI, Department of Electrical Engineering and Information Technologies of the same University) and Dr. Antonio Saverio Valente (post-doc of DIETI). This is an innovative system for supporting complex clinical decisions based on artificial intelligence. The Device is a system based on hardware components that processes and manages in real time all information of a chronic patient, also evaluating global severity indices, enucleated from elementary parameters characteristic of the pathology or the poly-pathological set (settable).

In the medical-health field are usually used medical records in paper format (usually residing in the hospital structures of competence) and only recently, and in some specific cases, the electronic record of the patient (electronic medical record). However, these tools do not allow the doctor to obtain synergic and comprehensive information, and, therefore, the overall concise and efficient assessment of the patient’s health and risk state, especially in the case of chronic polypathological patients, where the risk of aggravations due to complications, and the risk of death, it is very high.

In the following, we report a literature review of the state of the art related to the field of application of the invention.

- Kasabov et al. [1] propose a decision support system based on Bayesian techniques whose application is focused to the exploiting of genetic information for the diagnosis of some types of diseases. The idea is to extract relational rules among gene sets and clinical data in order to highlight the incipient onset of some types of diseases, that can be treated through customizable gene treatments.
- A system for supporting medical decisions is instead proposed in Schmidt et al. [2], where the proposed invention restricts the use of data mining techniques only to similarity analysis with respect to patient clusters.
- The application of fuzzy logic to medical decisions is instead proposed in Park et al. [3] where the method includes a multi-stage acquisition of clinical data but it is limited to a single target pathology, therefore it does not explicitly consider the coexistence of multiple pathologies and factors of high comorbidity.
- The creation of an automatic system for generating an Electronic Health Record (EHR), that integrates all the patient’s health data collected from public and private information systems, is the object of the invention by Vishnubhatla Suresh-Kumar Venkata et al. [4].

More classical approaches use decision support systems only as interfaces to access and update patient records.

- This kind of approach is for example followed by Mack et al. in [5] where the system is able in handling large amounts of clinical data, but not predicting patient status and risk analysis.
- Likewise, Tremper in [6] proposes a device for compiling the electronic medical record based on data from the acquisition of fundamental clinical variables.
- Randazzo et al. [7] instead focus on the generation of alarm signals but the tool uses very simple comparative methods that only verify if some temporal patterns of data exceed a priori pre-established thresholds.
- Lee et al. [8] instead propose the implementation of algorithms of semantics and ontology for the selection, extraction, and storage of information from data, also recorded with natural language.

The advantages of such a novel device are: i) limitlessness in the numbers of parameters that can be simultaneously evaluated and constantly monitored in their variations; ii) possibility of evaluating the patient’s response to a given pharmacological treatment to verify its effectiveness; iii) possibility of evaluating the patient’s response to eventual changes in his lifestyle.

## II. METHODOLOGY

In the invention, a dedicated hardware elaboration system manages automatically and processes in real-time all the patient’s clinical data and the main clinical variables for both diagnosis and treatment support, also detecting any shortcomings in analyzed data. For example, in the illustrative case of a patient affected by beta-thalassemia, by using the invention it is possible to optimize treatment scheduling defining precisely the date of the next transfusion. Or, if we consider the case of a patient with diabetes mellitus (DM), for whom the first cause of death is cardiovascular disease (MCV) when an early arteriosclerosis occurs, our invention can highlight to the clinician, thanks to specific alerts, the lack of the “value of ferritin in the blood” (identified as a significant cardiovascular risk index) by automatically analysing the data of the chronic patient and/or the presence of out-of-range clinical parameters, effectively supporting the doctor during the diagnostic or treatment prescriptions phases, thus preventing clinical risks.

**Fig. 1:**
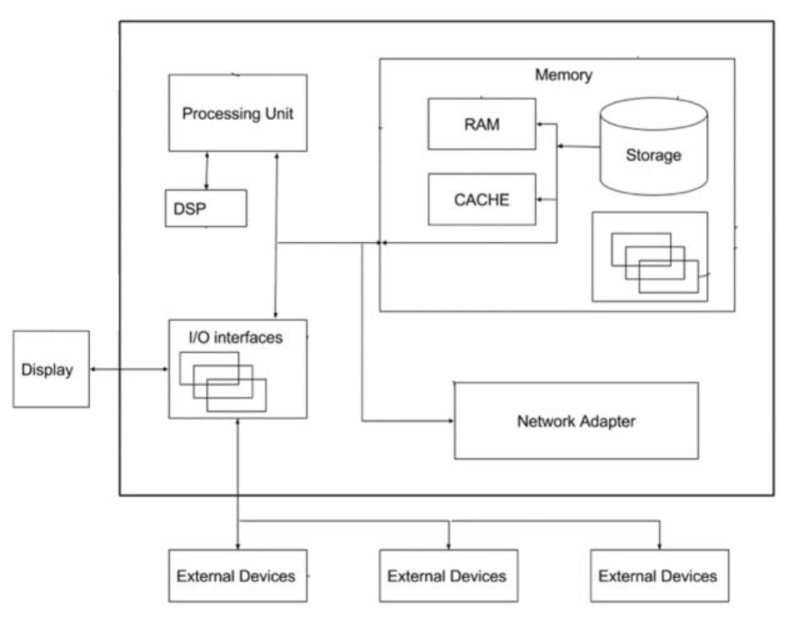
schematic view of the hardware realization of the equipment.

Figure 1 represents a rough schematic view of the hardware realization of the equipment and is one of the examples of implementation.

The device processes clinical data and allows, through continuous monitoring and computerized management of the state of health, the prevention of clinical risk in chronic polypathological patients, for whom no tools are currently available that allow an efficient assessment of the state of health and clinical risk.

The device looks like a portable on-chip container, scalable and accessible even remotely, providing the caregiver with a global and dynamic view of the patient’s clinical status according to elementary parameters that can be set by the doctor, with the additional advantage of being able to check the effectiveness of a drug treatment and / or the effect of a change in the subject’s lifestyle. The definition and evaluation of personalized indicators relating to the severity of the clinical condition of the chronic polypathological patient also allows the establishment and setting of specific alarm thresholds relating to the various pathologies affecting the individual and consequently to correct the therapeutic approach based on the outputs provided from the device.

## III. RESULTS AND DISCUSSION

The device, easy to use for doctors, offers them a complete picture of the patient and the consequent possibility of defining a more appropriate diagnostic-therapeutic-assistance procedure. All this is strictly connected to an optimization of the costs and resources used in the mana5rfgement of this type of patients whose health status, due to the concomitance of different pathologies and the absence of specific indicators, proves to be difficult both to determine and to to monitor.

**Fig. 2:**
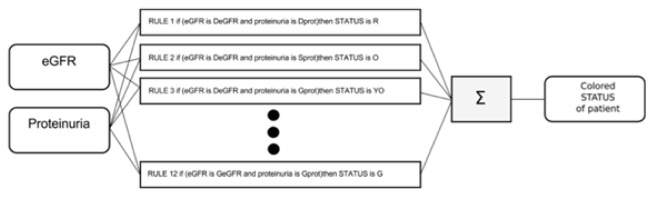
Entry-exit scheme.

The following is an elementary example that contemplates the use of only two input variables, identified in “proteinuria” and in the “glomerular filtration rate” (eGFR), fundamental for monitoring the progression of chronic renal failure, and of a global index representative of the degree of severity of the disease itself (see figure 2 for the entry-exit scheme).

**Fig. 3:**
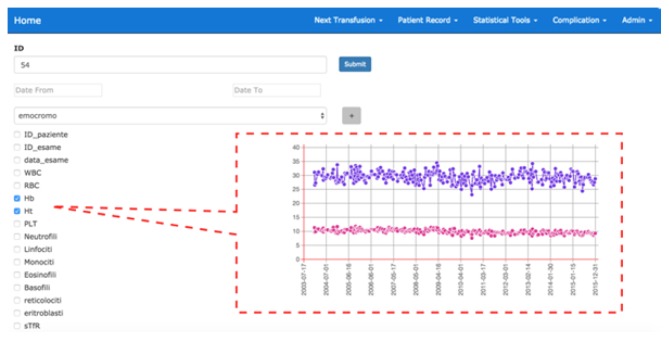
Example of monitoring the temporal trend of two parameters (Hb and Ht) related to the blood count of a generic patient.

This decision support system also allows the clinician to explore patient data or suitably clustered patient groups, selecting the parameters to be monitored and the type of representation (which can for example be graphic / tabular). Figure 3 shows an example of monitoring the temporal trend of two parameters (Hb and Ht) related to the blood count of a generic patient.

**Fig. 4:**
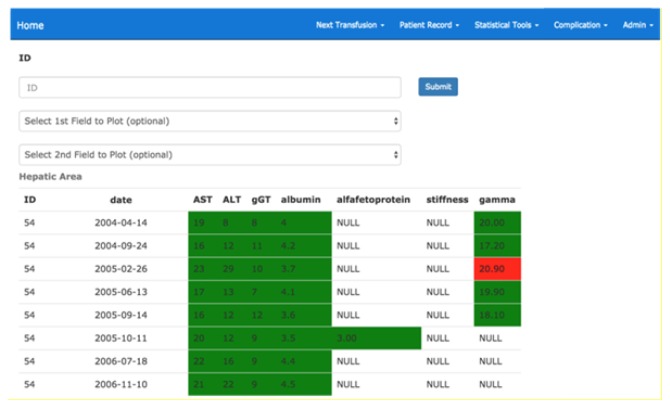
Summary picture for the kidney area related to a patient with chronic kidney disease.

The system also provides for the possibility of showing summary dashboards divided by area (liver, cardiac etc.) containing the most significant coloured parameters according to the belonging range and the display of alert messages in the case of values higher than the critical thresholds established in relation to the patient’s pathology and characteristics (gender, age etc ..). Figure 4 shows an example of a summary picture for the kidney area related to a patient with chronic kidney disease.

To sum up, hte invention has the advantage of:

- Offer a complete and easy to understand the picture of the clinical status of the polypathological patient;
- Allowing the real-time automatic monitoring of the patient’s health status;
- Optimize the treatment schedule, defining in a more accurate and customizable way, the most appropriate date for the next visit/treatment on the basis of the inference results of all clinical and diagnostic data;
- Optimize clinical activities in terms of costs, the duration of the visits and related efforts;
- Produce visual alerts at the occurrence of out of range events in the clinical parameters or in severity index;
- Generate automatically customized global indicators related to the severity of a disease and its comorbid factors, thus providing to the physician quick assessment of the patient’s clinical status and the precise suggestion about results coming from therapeutic actions pursued or to be pursued;
- Support the doctor in the definition of any corrective action (e.g., to dose increase/reduction of a given drug);
- Provide tools for statistical analysis, prediction, and visualization of any trend related to the characteristic parameters of the pathology and their possible correlations;
- Cluster the patients’ cohorts on the base of meaningful parameters (settable by users) for the definition of groups of patients with similar behaviours, that hence can be also exploited as benchmarks during diagnosis and decision making processes.

The use of calculation systems that operate with a logic that emulates the evaluation process of the physician, but at the same time guarantees the objectivity of the result that is not influenced by external factors, allows saving the time and the energy involved into the decision-making process.

A further purpose of the device is the aid to the research activities since the system allows the researcher to easily and in real time study the clinical data of a single patient in order to obtain feedback on the treatments performed with respect to suitably selected and clustered patient groups. In fact, it provides tools for statistical analysis, data mining and fuzzy logic, for the automatic extraction of correlations between all or part of the monitored parameters, allowing the extraction of implicit information in the data and significant patterns.

## IV. CONCLUSION

The creation of this promising decision-making tool is one of the numerous demonstrations of how the intuition and experience of the experts of the Public Health Department of University of Naples Federico II, who has always been committed to health prevention, medical-scientific research and the interdisciplinary management of health activities, together with the opportunities offered by technological and digital progress, can expertly translate into an innovative product, concrete but above all responding to a complex and real clinical need.
